# Reported Adverse Events Following SARS-CoV-2 Vaccinations in the Canadian Province of Alberta and Associated Risk Factors: A Retrospective Cohort Study

**DOI:** 10.3390/vaccines12121409

**Published:** 2024-12-13

**Authors:** Yei Mansou, Mahalakshmi Kumaran, Gregory Farmer, Kyle Kemp, Hussain Usman, David Strong, George K. Mutwiri, Khokan C. Sikdar

**Affiliations:** 1 School of Public Health, University of Saskatchewan, 104 Clinic Place, Saskatoon, SK S7N2Z4, Canada; yem505@usask.ca (Y.M.); george.mutwiri@usask.ca (G.K.M.); 2 Public Health Surveillance and Informatics, Provincial Population and Public Health, Alberta Health Services, 10301 Southport Rd., Calgary, AB T2W1S7, Canada; mahalakshmi.kumaran@albertahealthservices.ca (M.K.); khokan.sikdar@albertahealthservices.ca (K.C.S.); 3 Provincial Population and Public Health, Alberta Health Services, 10301 Southport Rd., Calgary, AB T2W1S7, Canada; hussain.usman@albertahealthservices.ca (H.U.); david.strong@albertahealthservices.ca (D.S.); 4 Community Health Sciences, Cumming School of Medicine, University of Calgary, 3330 Hospital Dr., Calgary, AB T2N4N1, Canada

**Keywords:** COVID-19, SARS-CoV-2 vaccines, adverse events, AEFI, ImmARI

## Abstract

**Background/objectives**: Coronavirus-19 (COVID-19) vaccines represent a significant milestone in the fight against coronavirus disease. Ongoing post-marketing surveillance and research are crucial for ensuring vaccine safety and effectiveness, aiding public health planning. **Methods**: Our retrospective cohort study included Albertans five years and older and vaccinated with at least one dose of an approved COVID-19 vaccine between 14 December 2020 and 30 April 2022. This epidemiological study aimed to determine the incidence of reported adverse events following immunization (AEFI) in Alberta and identify associated risk factors. **Results**: The study included 3,527,106 vaccinated Albertans who met the study inclusion criteria. A total of 2541 individuals (72.0 per 100,000) reported an AEFI, with 2759 adverse events, most of which occurred following the first dose of vaccine and within the first week post-vaccination. Of these, 70.4% were female, and the highest incidence was in the 35–54 age group. Given that mRNA vaccines were predominantly administered across Canada, we report AEFI rates (per 100,000 doses) for the mRNA vaccine brands at 27.7 for Pfizer and 40.7 for Moderna. Allergic events were the most frequently reported AEFI, followed by adenopathy. Logistic regression analysis indicated that sex (with females at higher risk), presence of comorbidities, days to symptom onset, vaccine type (mRNA vs. mixed doses), and the number of doses were significant factors associated with an AEFI event. **Conclusions**: Our study provides valuable information to guide policies surrounding COVID-19 vaccination. While the risk of serious adverse events was low in the population-based sample, further research is warranted to identify and investigate other possible risk factors that are still unknown.

## 1. Introduction

The Coronavirus-19 (COVID-19) pandemic was one of the greatest threats to national health in Canada. There have been a total of 4,617,095 confirmed cases, 51,720 deaths, and a mortality rate of 135.23 (per 100,000 persons) (as of 10 March 2023) [1]. Compared to other rich nations of similar size in the Group of 7 (G7) nations which administered the same vaccines, Canada fared better when comparing the rate of mortality, which is plausibly related to the strict public health policies Canada instituted throughout the pandemic, along with highest vaccine uptake in the general population of approved vaccines [2]. Though the same study used ecological analyses, national surveillance in Canada using aggregated individual data observed better health outcomes, including decreased number of confirmed cases, lower hospitalization, and mortality rates in vaccinated compared to those who chose to remain unvaccinated [3].

On 9 December 2020, Canada authorized its first COVID-19 vaccine, the Pfizer-BioNTech COVID-19 vaccine. Following Pfizer approval, on 23 December 2020, the Moderna COVID-19 vaccine was also approved for use in Canada. The Pfizer-BioNTech COVID-19 vaccine and Moderna COVID-19 vaccine are mRNA-based vaccines—the first of their kind. As of 16 March 2022, there are six COVID-19 vaccines approved for use in Canada, which include Pfizer-BioNTech Comirnaty, Moderna Spikevax, Janssen, AstraZeneca Vaxzevria, Novavax Nuvaxovid, and Medicago Covifenz [4].

Overall, mass vaccination efforts have limited adverse disease outcomes in the population, with 84.7% of the Canadian population five years and older receiving at least one dose of an approved vaccine (as of 3 December 2023) [5]. The approved COVID-19 vaccines in Canada were rigorously tested during vaccine development and have been reported to be safe and effective under the Health Agency of Canada [4]. However, understanding the risk of adverse events from vaccination in the population post-implementation is an important transparency metric that allows the public, researchers, and practitioners to be equipped with the benefit–risk tradeoff. Therefore, post-marketing surveillance of vaccine safety can provide room for further vaccine uptake in the population by inspiring public confidence.

With every vaccine, there is potential for adverse events following immunization (AEFI), and the COVID-19 vaccines are no exception. An adverse event may be any unfavorable or unintended sign, abnormal laboratory finding, symptom, or disease [6]. In Canada, a total of 54,569 AEFIs have been reported as of 3 March 2023, and of the reported AEFIs, 43,844 were deemed non-serious, while 10,685 were deemed serious reactions [7]. The Public Agency of Canada has reported conditions of AEFI associated with COVID-19 vaccines, including paraesthesia, vaccination site pain, headache, pruritus, dyspnea, pruritus, and fatigue [7]. More serious reported AEFIs include myocarditis, pericarditis, Bell’s Palsy, blood clots with low platelets, capillary leak syndrome, and Guillain–Barré syndrome (GBS) [7]. Similar patterns of AEFI’s have been observed in other countries. A systematic review conducted by Al-Ali et al. reported 2567 cases of adverse events following various COVID-19 vaccines (including Pfizer and Moderna), which included hematological, cardiac injury, myocarditis, myopericarditis, and myocardial infarction adverse events [8]. Similarly, authors Lane et al. observed 18,204 reports of myocarditis and pericarditis following mRNA vaccination in the USA, UK, and Europe [9].

While there are several government agency monitoring reports available online regarding AEFIs in Canada both nationally [10] and sub-nationally [11,12,13], there remains a lack of published surveillance literature on the topic. To our knowledge, one study has examined AEFI in the Canadian context, which investigated AEFIs within 7 days of vaccine administration [14]. The same study did not breakdown AEFIs by Canadian provinces, as it used data from a national surveillance effort. This limits the ability of provinces to gauge public health progress, as healthcare and public health decision making in Canada is at the discretion of provincial jurisdictions.

The current study will build upon this limitation by reporting adverse events at a provincial level. Furthermore, although there are studies reporting adverse events following COVID-19 vaccination, the associated risk factors still need to be fully understood in the Canadian context. Therefore, the current study aims to determine the incidence proportion and associated risk factors of AEFIs among individuals five years and older living in Alberta, Canada, following COVID-19 vaccination within 42 days of observation.

## 2. Materials and Methods

### 2.1. Study Design and Population

A retrospective cohort study design was adopted to review and analyze the data from administrative health datasets to determine the incidence and incidence proportion of adverse events reported up to 42 days following SARS-CoV-2 vaccination. The study examines various factors linked to AEFI events, including demographics, SARS-CoV-2 vaccine brands, and vaccination dose series, to determine if these characteristics are associated risk factors for AEFI. We included Albertan residents aged five years old and above who received at least one dose of an approved COVID-19 vaccine between 14 December 2020 and 30 April 2022. Exclusions were non-Albertan residents and/or individuals under five years of age, those who received non-approved vaccines, or vaccinations outside the study period.

The study was approved by the Conjoint Health Research Ethics Board (CHREB) at the University of Calgary and the Research Ethics Board (REB22-0525) at the University of Saskatchewan. Since it is a population-based study, the ethics board waived the requirement of written informed consent from the study participants.

### 2.2. Data Source(s)

For the study duration between 14 December 2020 and 30 April 2022, we accessed de-identified records related to immunization status, the number of doses administered, the vaccine brand administered, and the date of each immunization. In addition, we obtained de-identified records for a documented AEFI event following a COVID-19 vaccine dose. The data include the types of adverse events, the individual’s age and sex, their hospitalization and emergency room visit status, the specific brand of SARS-CoV-2 vaccine received, and the number of vaccine doses administered. Records were linked together using an individual’s unique lifetime identifier (ULI) by qualified Alberta Health Services (Alberta’s single health authority) staff, and data were de-identified for study investigators prior to release.

All the records for immunizations were accessed from the provincial clinical information system (Meditech) that delivers and supports a range of applications, allowing for a shared, province-wide electronic health record [15]. All reported adverse events following any vaccinations in Alberta are recorded in the Immunization and Adverse Reaction to Immunization (ImmARI) database. ImmARI is a central repository maintained by Alberta Health (Alberta’s health ministry), which maintains a list of administered vaccines and reactions to vaccines within the province. All health practitioners are required to submit immunization and assessment event information to ImmARI. This is facilitated through a standardized online questionnaire filled out by the healthcare practitioner and closely aligns with national reporting guidelines [16]. Health practitioners who do not have an electronic medical record system capable of integrating with ImmARI can report all immunizations and associated assessments through a web application called the immunization direct submission mechanism (IDSM). The IDSM facilitates the electronic submission of immunization and assessment events into ImmARI [17]. In cases where practitioners could not fill out the form, a central telephone line was available to facilitate central AEFI reporting to the relevant health authority [16].

To calculate the presence of comorbidities in patients with an AEFI, we extracted information from the following administrative health databases: the Discharge Abstract Database (DAD) for hospital admissions, the National Ambulatory Care Reporting System (NACRS) for emergency department visits, and physician billing claims for physician visits. We used a lookback period of 24 months prior to April 2022. A full list of ICD codes describing the comorbidities used in the extraction algorithm is available elsewhere [18].

Based on the patient’s postal code, health information was geocoded to one of the five Alberta Health Services geographical zones (AHS zones): South, Calgary, Central, Edmonton, and North zones. Reporting by zones enables local decision-making and enhances the capability of the organization to respond to local communities, staff members, patients, and clients [19]. This allows outcome estimates for incident proportion calculations to be linked to AHS zones, sex, and age-specific demographic groups. Population estimates were retrieved from the Alberta Health Care Insurance Plan (AHCIP) population registry files (mid-year adjusted) available through the Government of Alberta [20].

### 2.3. Risk Factor Variables

Risk factor variables in logistic regression models were developed using a pre-established algorithm for high-risk comorbidities likely to be associated with COVID-19 disease [18] and aligned with the Centre for Disease Controls review of high-risk medical conditions and severe COVID-19 infections [21]. Briefly, high-risk pre-existing condition categories included respiratory diseases, heart disease, cerebrovascular disease, metabolic diseases, and other diseases (e.g., clinical obesity or organ transplant) [18]. If an individual had at least one condition identified in an administrative health database, they were defined as having a comorbidity. Other risk factor variables of interest were retrieved from ImmARI and included age (in years), days from immunization to onset of symptoms (in days), sex assigned at birth (male, female, and unknown), number of vaccine doses received, date of adverse event, and vaccine type (mRNA, mixed doses, and non-mRNA).

### 2.4. Outcome(s)

The main outcome of interest for the current study was an adverse event. Adverse events are defined as, “any untoward medical occurrence following immunization which does not necessarily have a causal relationship to the vaccine. The adverse event may be any unfavorable or unintended sign, abnormal laboratory finding, symptom or disease” [6]. Serious AEFIs can be defined as, “an event that results in death, hospitalization or prolongation of an existing hospitalization, persistent or significant disability or incapacity, congenital anomaly/birth defect or is life-threatening or is a medically important event or reaction” [6]. AEFIs that do not meet the definition of a serious event are classified as non-serious [22]. The definition and criteria for AEFIs were adapted for the current study and aligned with national guidelines based on expert input, research, and product monographs [16]. A full list of conditions outlined by the Public Health Act of Alberta is available elsewhere [16].

Adverse Events of Special Interest (AESI) may or may not be causally associated with the vaccine product, and they need to be meticulously monitored and studied [23]. Some of the designated AESIs are myocarditis, multisystem inflammatory syndrome in children, acute respiratory distress syndrome, single-organ cutaneous vasculitis, and acute kidney injury. The definition of AESI was also adapted for the current study. A full list of conditions for AESI is available elsewhere [23].

Other severe or unusual events refer to events of unknown etiology occurring within four weeks of immunization that are of epidemiological significance, generally requiring medical intervention, are life-threatening and require hospitalization, are fatal events, or result in a residual disability that is not covered by other AEFI categories [16]. Narcolepsy and shoulder injury related to vaccine administration site are also reported under this category.

### 2.5. Statistical Analysis

Descriptive analyses included the percentage of the vaccinated population and incidence proportion (per 100,000 persons) of reported AEFI by sex, age, zone, and vaccine brands. The statistical significance of incidence proportion across groups was estimated using chi-square tests. The majority of the current study findings and corresponding statistical analysis are performed based on AEFI cases with at least one dose of an mRNA vaccine, as they accounted for the majority (96.35%) of the total administered doses in the study sample. The rate of AEFI per 100,000 doses administered was calculated for each vaccine brand to limit the double counting of patients who received a different number of vaccine doses.

Logistic regression analysis was conducted to determine risk factors for our study’s outcome measures. This study focused on the two most frequently reported AEFI outcomes: allergic events (reported in 30% of AEFI patients) and adverse events of special interest (AESI, reported in 13% of AEFI patients). We developed two independent logistic regression models; in model 1, the outcome variable was allergic events; in model 2, the outcome variable was adverse events of special interest. Both models were adjusted for age, presence of comorbidities, days from immunization to the onset of symptoms, sex assigned at birth, number of doses received, and vaccine type. The odds ratio (OR) and associated 95% confidence intervals (CIs) were reported. Statistical significance was evaluated against a *p*-value ≤ 0.05. All statistical analyses for this study were conducted using IBM SPSS Statistics for Windows (Version 25), Armonk, NY, USA [24].

## 3. Results

### 3.1. Baseline Characteristics

**Figure 1:**
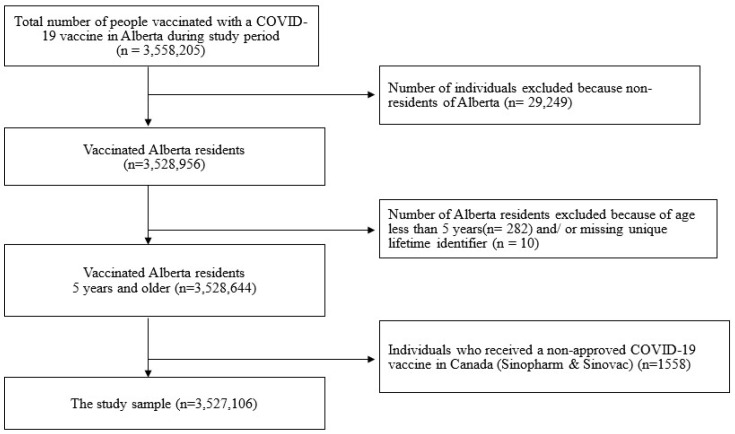
Study sample determination from the ImmARI Meditech databases (n = 3,527,106).

A total of n = 3,558,205 were vaccinated for COVID-19 over the study period. During our study period between 14 December 2020 and 30 April 2022, a total of 84.2% (n = 3,527,106) of Alberta residents 5 years and above had received at least one dose of an approved COVID-19 vaccine (Figure 1). A total of n = 31,099 were excluded from the sample as they did not meet the inclusion criteria.

The highest vaccination rate was observed among Albertans 65 years and older, with 97.0% receiving at least one dose (Table 1). Followed by the 55–64 age group with the second-highest vacation rate of 91.3%. The lowest vaccine uptake was observed in the youngest group (5–17 years of age), with 61.7% receiving at least one dose. Females (85.2%) had slightly higher vaccine uptake than males (83.1%). Similarly, there was a difference in the distribution of vaccine uptake across the five AHS zones. The Edmonton zone had the highest percentage (87.5%) of vaccinated Albertans, followed by the Calgary zone (86.9%), the South Zone (79.9%), and the Central Zone 76.2%. The North zone had the lowest percentage (75.6%) across all groups (Table 1).

In Alberta, a total of 8,613,563 doses of vaccines were administrated (Table 2). Among the different vaccine brands, Pfizer doses accounted for most of the doses administrated to the public (76.3%), followed by Moderna Spikevax (20.0%) and AstraZeneca Vaxzevria (3.5%). Janssen and Novavax Nuvaxovid combined accounted for only 0.11% of vaccines administered. Within ImmARI, there was no record of administered dose(s) for Alberta’s Medicago Covifenz COVID-19 vaccine during the study period (Table 2).

### 3.2. Overall Rate of Adverse Events

The study observed 3,527,106 vaccinated Alberta residents. Amongst the vaccinated individuals, a total of 2541 individuals reported 2759 adverse events following COVID-19 vaccination (Table 1). The overall incidence proportion of AEFI following COVID-19 vaccination is 72.1 cases per 100,000 vaccinated individuals. It is important to note that the same individual may experience multiple adverse events after receiving a vaccination. This is reflected by the larger number of adverse events amongst a smaller number of individuals.

In our study, about 70.4% of reported AEFI cases were female, with the highest rate of 100.7 AEFI events, compared to males, with a rate of 42.9 AEFI events per 100,000 vaccinated individuals (Table 1). Considering the AEFI events across different age groups, irrespective of the brand of COVID-19 vaccine given, the highest percent (39.1%) of AEFI was noted in the 35–54 age group at a rate of 90.6 per 100,000. Among the 2759 reported adverse events, 1117 (40.4%) individuals required treatment in an emergency room (ER), 227 (8.2%) were hospitalized, and 1415 (51.2%) of the adverse events did not require medical treatment. All AEFIs were reported by physicians or care teams and suggest that a medical condition may not have been severe enough to require treatment in an emergency room or inpatient services.

### 3.3. Adverse Events by Vaccine Manufacturer

A higher proportion of AEFI events was reported among individuals who received the Pfizer vaccine (66%), which corresponds with the approximately 6.5 million Pfizer doses administered in the province (Table 1). Similarly, Moderna, the second most administered vaccine, accounted for the second highest percentage of AEFI events at 24.6%, followed by AstraZeneca/Vaxzevria at 8.5% and Janssen at 0.3%. When considering the rate of AEFI per 100,000 doses administered for each manufacturer, AstraZeneca/Vaxzevria had the highest rate at 76.7 AEFI events per 100,000 doses. This was followed by Janssen with 66.1, Moderna with 40.7, and Pfizer with the lowest rate at 27.7 AEFI events per 100,000 (Table 2).

Most COVID-19 vaccine doses administered in Alberta were mRNA vaccines (96%), which corresponds to 98% (n = 2519/2759) of adverse events associated with the use of Pfizer and Moderna vaccines. Of all the AEFI events reported in the province, approximately 66% (n = 1818 events reported) were attributed to Pfizer, while 25% (n = 701 events were reported as Moderna. Therefore, the remainder of this study will concentrate on analyzing adverse events specifically following mRNA vaccination.

#### 3.3.1. AEFI Events Following mRNA Vaccines

**Figure 2:**
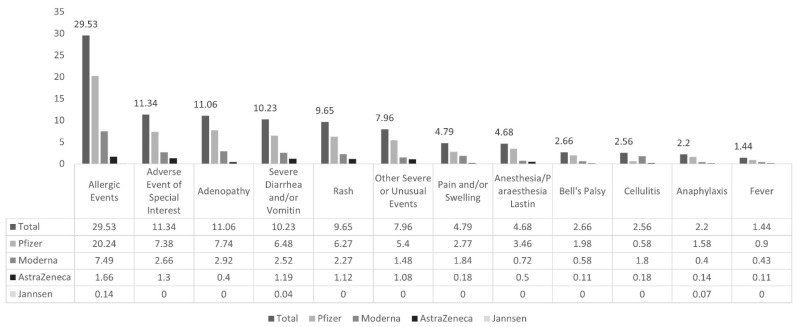
Percent frequency of total AEFI for different types of AEFI events (n = 2759) by vaccine manufacturer.

The proportions of different types of adverse events reported in our study cohort are shown in Figure 2. The top five most common were allergic events (29.5%), followed by adverse events of special interest (11.3%), reactions with adenopathy (11.0%), severe diarrhea (10.2%), and rash (9.6%). Less common events (between 1 and 7 percent) included other severe or unusual events, pain and/or swelling, anesthesia/paraesthesia lasting 24 h or more, Bell’s palsy, cellulitis, anaphylaxis, and fever. The least common types of AEFI events (less than 1% of cases) included thrombocytopenia, Guillain–Barre syndrome, nodule, erythema multiforme, encephalitis, acute disseminated encephalomyelitis (ADEM), myelitis, convulsion/seizure, arthralgia/arthritis, meningitis, and infective abscess (Table S1).

#### 3.3.2. AEFI Onset in Days and Dose Series

**Figure 3:**
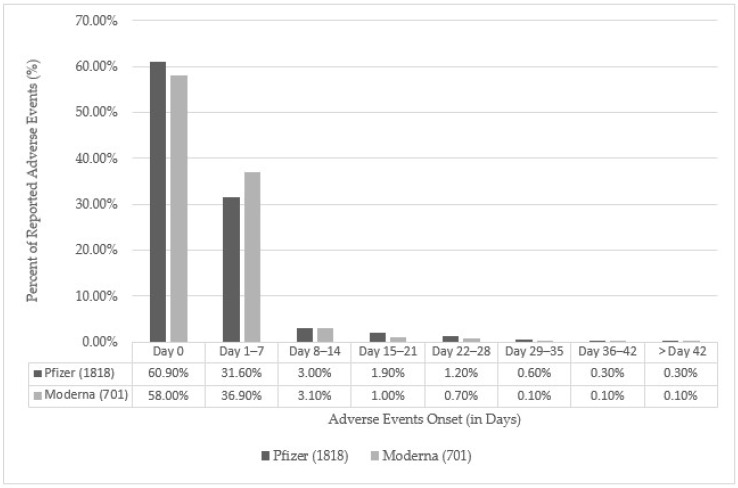
Days to onset of AEFI following mRNA vaccine administration of 2519 individuals. Notes: Of the 1818 reported adverse events for Pfizer, 3 adverse events did not have available onset days and, thus, were excluded from the above graph.

Most of the adverse events reported for Pfizer and Moderna occurred within the first seven days following vaccination. About 60.9% (Pfizer, n = 1108) and 58.0% (Moderna, n = 405) of events occurred less than 24 h after vaccination (Figure 3). The remaining AEFI events, 31.6% for Pfizer (n = 574) and 36.9% for Moderna (n = 259), were recorded between days 1 and 7. The remaining events (about 9%) occurred between days 8 and 42 (Figure 2). A small proportion of events (0.3%) involving 5 cases of Bell’s Palsy were reported after 42 days for Pfizer; this timeline is well within the recommended Alberta Health reporting guidelines [16], and one case of AESI was reported for Moderna on day 43, which is one day over the reporting window for AESI. However, under the recommendation of Alberta’s medical officer of health, an exception for this case was made and was considered an AESI. Of the 1818 reported adverse events following Pfizer vaccination, three reported adverse events did not have information on the date of symptom onset and were not included in Figure 2.

During the observation period, the proportion of AEFI events was higher following the first dose of the vaccine compared to subsequent doses (doses ≥ 2) for both Pfizer and Moderna. Among the 1818 AEFI events that were reported after receiving the Pfizer vaccine, 69% (n = 1247) occurred following the first dose, while 31% (n = 571) occurred following doses ≥ 2 (with significant chi-square test [*p*-value < 0.0001]). Specifically, a higher proportion of adenopathy and allergic events were observed among patients who received a single dose of Pfizer compared to multiple doses. A similar trend was observed for Moderna doses; 73% (n = 509) and 27% (n = 192) of the reported adverse events following the first dose and subsequent doses (≥2) of the Moderna vaccine, respectively (Table S2). Compared to allergic events, a higher proportion of AESI was observed among individuals who received multiple doses of Moderna (15.6%) (Table S2).

### 3.4. Multivariable Logistic Regression

The findings of model 1 (Table 3) indicate that individuals who received mixed doses (both mRNA and non-mRNA vaccines) had a significantly increased risk of experiencing an allergic event, with an odds ratio of 1.32 (95% confidence interval (CI): 1.07–1.62), indicating a 32% higher risk compared to those who received only mRNA vaccines for all doses. The most significant trend observed in the model points to the variables, days to symptom onset, male gender, and the second dose, which were protective factors against allergic events. Allergic events were frequently observed on the day of immunization. Furthermore, the lower incidence of allergic events among men compared to women (Table 1) is consistent with the trends observed in model 1.

In model 2, the results revealed that the presence of comorbidities significantly increases the odds of an AESI, with an odds ratio (OR) of 6.35 (95% CI: 4.42–9.12). Additionally, the number of doses was also a significant risk factor (OR = 1.63, 95% CI: 1.22–2.18). Male gender was associated with higher odds of an AESI, with an OR of 4.09 (95% CI: 3.13–5.35). Furthermore, individuals who received mixed doses (both mRNA and non-mRNA vaccines) had increased odds of an AESI (OR = 2.00, 95% CI: 1.44–2.77) (Table 3).

## 4. Discussion

The manufacturing and approval of COVID-19 vaccines, during an unprecedented time in public health, was a significant step in controlling the negative consequences of the coronavirus disease in the population. However, adverse events following vaccination with different brands of COVID-19 vaccines are well-documented in the literature and were observed in the current study. Our study assessed the incidence of AEFI cases, frequency of occurrence of adverse events by dose series, onset interval of adverse events, and the associated risk factors for the residents of Alberta who were five years and older and received at least one dose of the approved COVID-19 vaccine during our study period.

Our study investigated the top five adverse events reported following Pfizer and Moderna COVID-19 vaccines. They were allergic events, AESI, adenopathy, severe diarrhea and/or vomiting, and rash. Other severe and unusual events, such as anesthesia and paraesthesia, anaphylaxis, fever, and Bell’s palsy, were also reported, though the incidence was higher following the Pfizer COVID-19 vaccine than the Moderna vaccine. In comparison, a systematic review by Dighriri et al. observed 10,632 individuals and reported the average most frequent side effects following Pfizer COVID-19 vaccination [25]. In the same study, side effects were injection site pain, fatigue muscle pain, local swelling, headache, joint pain, chills, fever, itching, lymph node swelling, nausea, dyspnea, and diarrhea; however, side effects were often mild, self-limited, and usually began within 24 h post-vaccination; anaphylactic shock or severe reactions were rare [25].

In our study, overall AEFI counts, including allergic events and adenopathy, were higher in female patients than male patients (Figure S1A,B). These were observed in individuals who were given the non-bacterial and viral COVID-19 vaccines (Pfizer and Moderna). This observation may reflect sex-based immunological differences demonstrating the higher immune responses to vaccines in women than in men. According to a review by Klein and Flanagan, women have higher antibody responses post-vaccination compared to men while also having more frequent reactions post-vaccination [26]. This observation has also been observed in studies from Japan [27] and surveillance reports in Canada [11]. Regardless of the mechanism of action underpinning higher reactions in females, we recommend data reporting and follow-up for AEFIs of any vaccine be sex stratified to garner insights into this phenomenon.

Other documented adverse events in the literature following COVID-19 vaccination were anaphylaxis, myocarditis, pericarditis, thrombosis with thrombocytopenia, cerebral venous sinus thrombosis, venous thromboembolism, and GBS [28,29,30,31,32,33,34,35,36,37]. Thrombocytopenia, GBS, encephalitis, acute disseminated encephalomyelitis, and myelitis were infrequent among the AEFI cases in the current study. Four cases of thrombocytopenia were reported following the Moderna COVID-19 vaccination, and 12 cases were reported following the Pfizer COVID-19 vaccination. In the literature, thrombocytopenia was frequently reported post-vaccination with AstraZeneca and Janssen COVID-19 vaccines, whereas only a few cases were reported after administering mRNA COVID-19 vaccines [28,30,35,37]. Furthermore, in the literature, thrombocytopenia is commonly reported in females younger than 60 years of age. Though thrombocytopenia was infrequently encountered in our study, the documented reports were higher in males (12 events) than in females (4 events). Oral contraceptives, contraceptive vaginal rings, hormone replacement therapy, and obesity are considered predisposing factors for the higher incidence of thrombocytopenia in females. Individuals with a history of venous thromboembolism, irrespective of sex, were also considered risk factors in the literature [30,36,37]. Explanatory reasons for this observation in the current study remain unclear.

In the literature, there is mixed evidence regarding days to onset of adverse events following immunization. Onset typically occurs within 1 week for certain adverse events. At the same time, other adverse events were reported to have an onset greater than 1 week. For example, injection site pain, fatigue, muscle pain, local swelling, headache, fever, lymph nodes swelling, and nausea were among some of the commonly reported symptoms 24 h following Pfizer vaccination [25]. In longer observation periods, authors Chen et al. observed venous thromboembolism onset was primarily within 5–42 days post-vaccination, with no reported cases within 1–2 days or greater than six weeks post-vaccination [30]. Continued monitoring is required in the Canadian population to determine reaction onset following vaccination for specific adverse reactions.

Evidence is also mixed for reports of adverse events following vaccination dose series. Some studies report most of the adverse events occurring following the first dose, while other studies observed adverse events primarily following the second dose. For instance, authors Matta et al. observed 69 cases of myocarditis post-mRNA COVID-19 vaccination in their study, and 11.5% of the patients developed symptoms after the first dose, with 88.5% developing symptoms after the second dose [38]. Another study by Ismail and Salama reported 32 cases of CNS demyelination following various COVID-19 vaccinations, including the COVID-19 mRNA vaccines, and 71.8% of the cases occurred post-first dose of the vaccine with an exhibition of neurological symptoms after nine days on average [39]. In the current study, adverse events primarily followed the first dose for both Pfizer and Moderna, demonstrating a slight dose–response relationship. This pattern was also observed in model 2 of the logistic regression analysis. Receiving two or more doses compared to just receiving one dose of an approved COVID-19 vaccine was associated with an increase in the odds of AESI but decreased the odds of an AEFI. One speculative reason for a dose–response relationship is the extent of prior SARS-CoV-2 infections and, particularly, the severity of the infection prior to receipt of a vaccine. For instance, a large prospective Canadian study following over 680,000 vaccinations observed that individuals with prior positive SARS-CoV-2 tests and severe symptoms were at greater risk for adverse events following receipt of the first dose of vaccination compared to no infection prior to dose administration [14]. The same study observed a dose–response relationship between the primary and secondary doses of BNT162b2 (Pfizer) and mRNA-1273 (Moderna) vaccines, with substantial attenuation of adverse events following the second dose; more specifically, mRNA-1273 was observed to have significantly lower odds of a serious health event amongst individuals with prior history of asymptomatic/mild COVID-19 infections. This phenomenon warrants further investigation in the Canadian population and requires further surveillance studies to confirm.

Compared to peer provinces, Alberta appeared to have lower rates of AEFI (per 100,000 doses). For instance, the Saskatchewan health authority reported a total AEFI rate of 45 per 100,000 doses administered, with a total of 1407 reports of AEFI, and 1304 were deemed non-serious [13]. Public Health Ontario released a report detailing AEFI over a 4-year observation period from December 2020 to May 2024, which reported 22,129 non-serious AEFIs, 1286 of which were serious AEFIs and 1774 were AESIs [11]. In the same report from Ontario, a total AEFI rate of 57.8 per 100,000 doses administrated was observed [11]. In a smaller province, Nova Scotia, an online report detailed a total AEFI rate of 39 per 100,000 doses administered [12]. One plausible explanation for this phenomenon is the marginal age and sex differences between provincial populations, which may moderate the effect on adverse reactions [10]. For example, the median age of Ontario is slightly older than Alberta, both pre- and post-pandemic and it is different between men and women [40]. While the distribution of age across age-specific strata is flatter in Ontario for 30–59-year-olds compared to Alberta, Ontario has a higher proportion of the population over the age of 65 overall [40]. Another reason could be the difference in population multimorbidity and the odds of severe reactions from a COVID-19 vaccine. According to one Canadian study using a national survey, the residents from some cities within the province of Ontario (excluding Toronto) were, on average, at higher odds of having multimorbidity’s compared to residents from Alberta when controlling for neighborhood residence and individual-level confounders [41]. In the current study, having at least one comorbidity was associated with increased odds of an AESI and lower odds of an allergic event. The Alberta population, compared to peer provinces, may, on average, have fewer multimorbidity’s and, therefore, have fewer AEFI and AESI events overall.

Vaccine safety and monitoring are paramount to ensure the vaccines remain safe and effective once administered to the population. Mass vaccination programs limit the rate of death and hospitalizations in the Canadian population and are one of a myriad of possible reasons Canada fared better during the COVID-19 pandemic compared to peer countries [2]. One Canadian study using data from the Canadian COVID-19 vaccination coverage surveillance system (CCVCSS) reported average weekly age-standardized incidence rate ratios in unvaccinated compared to vaccinated individuals who completed their dosage series in Canada between June 2021 and January 2022 [3]. The study reported incidence rate ratios of 7.1 to 21.0 for hospitalizations and 11.3 to 15.4 for mortality. That is, those unvaccinated were at least 7 times more likely to be hospitalized and at least 11 times more likely to die of the disease than those who were fully vaccinated [3]. Though non-causal, the implication is plausible that mass vaccination programs limit the harmful health outcomes in the population and are still one of the major defenses against virulent respiratory illnesses.

### Strengths and Limitations

This study’s fundamental strengths are, firstly, that it is based on a retrospective cohort study design with a lookback period from the arrival of the first SARS-CoV-2 vaccine (Pfizer) on 14 December 2020, in Alberta, Canada, to 30 April 2022. The cases of AEFI reflected in this report are only physician-verified and approved AEFI reports, limiting recall bias of reported events. Secondly, the results have provided important information that will further guide policies surrounding COVID-19 vaccination in Alberta and other parts of the world, given the study design and sample size that allows for the generalizability of study results. Finally, to our knowledge, this study will be the first peer-reviewed article that assesses the reported adverse events and associated risk factors following COVID-19 vaccination among residents of Alberta.

Despite the strengths of our study, there are a couple of limitations to note. First, the risk difference and the relative risk were not calculated due to the unavailability of an adequate number in the unvaccinated group to serve as an appropriate control. Second, while our study provides the list and overall count of adverse events expected to be documented under AESI and other severe or unusual event categories per the Alberta health immunization policy guidelines, our study cannot provide the specific AEFI types that were documented in these categories, as they were not accessible from the Ministry of Health. However, we have provided insight into the types and frequency of adverse events that reflect these categories. Third, while case reporting follows a standardized assessment process that aligns closely with national reporting guidelines [16,22], it does not apply causality assessment tools such as the World Health Organization—Uppsala Monitoring Centre (WHO-UMC) criteria or the Hallas criteria [42]. Adverse drug reaction assessment tools like the WHO-UMC causality assessment tool are used to determine the likelihood of a drug or biological agent producing an adverse outcome. The WHO-UMC criteria have six categories to classify the agent as certain, probable, possible, unlikely, conditional, and/or unclassifiable [43], while the Hallas criteria are split into four categories: definitely avoidable, possibly avoidable, unavoidable, and/or unclassifiable [44]. As the current study could not include these assessment protocols in its data collection or analysis, the generalizability of the findings to other countries or health jurisdictions may be hindered. Furthermore, we could not stratify our analyses by these causal categories; therefore, it is assumed the reported AEFI cases included could be those AEFI cases that do not meet the causality criteria to be defined as a case, or in the case of both assessment tools would be deemed as unclassifiable [16]. As such, caution is warranted when interpreting the results of the study. Finally, the utilization of passive surveillance systems for vaccine safety signals may be prone to selection bias if reactions are minor and self-limited (e.g., rash or injection site pain), as individuals may not seek medical attention. However, Alberta utilizes a universal healthcare model whereby any individual can access a basic standard of care at no extra cost. As such, the impact on the completeness of data in the current study on rare AEFI case reporting may be minimal.

## 5. Conclusions

The findings from this study of the Alberta population demonstrate growing evidence of the count and rate of adverse events following COVID-19 vaccines. In our view, the benefits of vaccination in the Canadian population far outweigh the risk of experiencing an adverse complication. Despite this, we recommend ongoing monitoring for rare diseases and research to investigate other possible risk factors and utilize the currently available evidence to guide COVID-19 vaccination policies.

## Figures and Tables

**Table 1:** Demographics and outcomes of patients with reported adverse events following SARS-CoV-2 Immunization (AEFI) in Alberta between 14 December 2020 and 30 April 2022 (n = 3,527,106).

		Population	Number of Vaccinated People (%)	Number of People with AEFI (%)	Rate of AEFI per 100,000 Population
**Total**		4,184,770	3,527,106 (84.2)	2541	72.13
**Age †**					
	5–17 years	715,208	441,585 (61.7)	143 (5.6%)	32.4
	18–34 years	1,031,620	870,281 (84.4)	621 (24.4%)	71.4
	35–54 years	1,252,172	1,096,470 (87.6)	993 (39.1%)	90.6
	55–64 years	546,087	498,491 (91.3)	412 (16.2%)	82.7
	65+ years	639,132	620,279 (97.0)	372 (14.6%)	60.0
	Unknown	-	-	-	-
Sex *					
	Male	2,100,928	1,746,372 (83.1)	750 (29.5%)	42.9
	Female	2,083,842	1,776,286 (85.2)	1788 (70.4%)	100.7
	Unknown Sex	-	4448	3 (0.1%)	-
**Zone**					
	South	293,456	234,525 (79.9)	233 (9.2%)	99.4
	Calgary	1,631,651	1,419,398 (86.9)	866 (34.1%)	61.0
	Central	449,902	342,629 (76.2)	260 (10.2%)	75.9
	Edmonton	1,366,305	1,195,374 (87.2)	934 (36.8%)	78.1
	North	442,789	334,846 (75.6)	248 (9.8%)	74.1
	Unknown	-	334	-	-

Notes: * Sex is reported as biological sex assigned at birth. † Age is in years.

**Table 2:** Distribution of vaccine doses, incidence proportion, and AEFIs for n = 8,613,563 COVID-19 vaccine doses administered in Alberta between 14 December 2020 and 30 April 2022.

		No. of Doses Administrated (%)	No. of AEFIs (%)	Rate of AEFI per 100,000 Doses Administrated
**All vaccines**	All	8,613,563	2759	32.0
**Vaccine brand**				
	Pfizer	6,574,260 (76.3)	1818 (66.0)	27.7
	Moderna	1,724,437 (20.0)	701 (24.6)	40.7
	AstraZeneca/Vaxzervria	303,763 (3.5)	233 (8.5)	76.7
	Janssen	10,592 (0.1)	7 (0.3)	66.1
	Novavax Nuvaxovid	511 (0.01)	-	-

Notes: All estimates in the table are rounded to the nearest tenth.

**Table 3:** Multivariable logistic regression analysis for allergic events and any adverse event of special interest (AESI) following approved COVID-19 vaccines in Canada.

	Model 1 Allergic Event (Yes) = 805 Other AEFI Event = 1928		Model 2 AESI (Yes) = 313 Other AEFI Event = 2420	
	OR (95% CI)	*p*-value	OR (95% CI)	*p*-value
**Age (in years)**	0.99 (0.99–1.00)	0.025	0.98 (0.97–0.99)	<0.0001
**† Symptom onset (in days)**	0.39 (0.35–0.44)	<0.0001	1.10 (1.08–1.12)	<0.0001
**Presence of comorbidities** Ref: No	0.81 (0.66–0.98)	0.029	6.35 (4.42–9.12)	<0.0001
**Doses ≥ 2** Ref: 1 dose	0.68 (0.55–0.85)	0.0005	1.63 (1.22–2.18)	0.0009
**Male** Ref: female	0.63 (0.50–0.78)	<0.0001	4.09 (3.13–5.35)	<0.0001
**Mixed vaccine type** Ref: mRNA	1.32 (1.07–1.62)	0.009	2.00 (1.44–2.77)	<0.0001
**Other vaccine type** Ref: mRNA	0.82 (0.28–2.41)	0.713	2.45 (0.81–7.41)	0.113

Notes: † Days from immunization to onset of symptom. Outcome variable model 1 is an ‘Allergic Event’ = 1 if patients have one or more approved events reported following vaccination (from dose 1 to dose 6). Model 2 outcome is an ‘AESI event’ = 1 if patients have one or more approved events reported following vaccination (from dose 1 to dose 6). The model is adjusted for age, sex (male, female, unknown), and vaccine type (mRNA = Pfizer or Moderna for doses 1 to 3; other = non-mRNA vaccine for doses 1 to 3; mixed = received both mRNA and non-mRNA vaccines for doses 1 to 3); onset days from dose administration to symptom onset; the presence of comorbidities up to 2 years prior to 30 April 2022.

## Data Availability

The datasets presented in this article are not readily available because they are restricted due to the confidentiality of patient’s health data. Requests to access the datasets should be directed to Alberta Health data stewards using health.inforequest@gov.ab.ca.

## Supplementary Materials

The following supporting information can be downloaded at https://www.mdpi.com/article/10.3390/vaccines12121409/s1, Table S1: Frequency of different types of AEFI’s by vaccine manufacturer. Table S2: Frequency of reported AEFI by dose and vaccine type. Figure S1: (A) AEFI type frequency by sex and by Pfizer, 14 December 2020 to 30 April 2022. (B) AEFI type frequency by sex and by Moderna, 14 December 2020 to 30 April 2022.
