# Supplementary material for: Reported Adverse Events Following SARS-CoV-2 Vaccinations in the Canadian Province of Alberta and Associated Risk Factors: A Retrospective Cohort Study
Source: Vaccines (Basel). 2024 Dec 13;12(12):1409. doi: 10.3390/vaccines12121409 (PMC11680294; doi:10.3390/vaccines12121409)
Supplement: Supplementary file 1 [file vaccines-12-01409-s001.zip › vaccines-3327045-supplementary.pdf]

*******SUPPLEMENTARY TABLES*******

Table S1. Frequency of different types of AEFI's by vaccine manufacturer.

Type of AEFI's	Total (%)	Pfizer (%)	Moderna (%)	AstraZeneca (%)	Jannsen (%)
Allergic Events	29.53	20.24	7.49	1.66	0.14
Adverse Event of Special Interest	11.34	7.38	2.66	1.3	0
Adenopathy	11.06	7.74	2.92	0.4	0
Severe Diarrhea and/or Vomiting	10.23	6.48	2.52	1.19	0.04
Rash	9.65	6.27	2.27	1.12	0
Other Severe or Unusual Events	7.96	5.4	1.48	1.08	0
Pain and/or Swelling	4.79	2.77	1.84	0.18	0
Anesthesia/Paraesthesia Lasting	4.68	3.46	0.72	0.5	0
Bell's Palsy	2.66	1.98	0.58	0.11	0
Cellulitis	2.56	0.58	1.8	0.18	0
Anaphylaxis	2.2	1.58	0.4	0.14	0.07
Fever	1.44	0.9	0.43	0.11	0
Thrombocytopenia	0.58	0.43	0.14	0	0
Guillain-Barre Syndrome	0.47	0.22	0.04	0.22	0
Nodule	0.22	0.07	0	0.14	0
Erythema Multiforme	0.18	0.11	0.07	0	0
Encephalitis, ADEM, Myelitis	0.18	0.14	0.04	0	0
Convulsion/Seizure	0.14	0.07	0.07	0	0
Arthralgia/Arthritis	0.07	0	0.04	0.04	0
Meningitis	0.04	0	0	0.04	0
Infective Abscess	0.04	0.04	0	0	0
Total	100	65.86	25.5	8.39	0.25

Table S2. Frequency of reported AEFI by dose and vaccine type.

	Pfizer			Moderna		
	N_1^P= **2626217**	$N_{\geq 2}^P$=**3949936**		N_1^M= **627070**	$N_{\geq 2}^M$=**1097749**	
	**Dose 1**	**Dose ≥2**	**P-Value**	**Dose 1**	**Dose ≥2**	**P-value**
**Total AEFI events (%)**	1,247 (69%)	571 (31%)	<0.0001	509 (73%)	192 (27%)	P < 0.0001
**Type of AEFI reaction, (%)**						
Adenopathy	98 (78.6)	117 (20.5)	< 0.0001	56 (11.002)	24 (12.500)	0.5778
Adverse Event of Special Interest	129 10.345)	74 (13)	0.0896	44 (8.644)	30 (15.625)	0.0071
Allergic Events	436 (35)	123 (22)	< 0.0001	148 (29.077)	57 (29.688)	0.8764
Anaphylaxis	32 (2.566)	11 (1.926)	0.3636	10 (1.965)	1 (0.521)	0.1570
Anesthesia/Paraesthesia	74 (5.934)	22 (3.853)	0.0766	16 (3.143)	4 (2.083)	0.4765
arthralgia/Arthritis	-	-	-	1 (0.196)	-	0.5355
Bell's Palsy	35 (2.807)	20 (3.503)	0.4184	12 (2.358)	4 (2.083)	0.8140
Cellulitis	8 (.642)	8 (1.401)	0.0849	40 (7.859)	10 (5.208)	0.2166

Convulsion/Seizure	2 (.160)	-	0.2850	1 (0.196)	1 (0.521)	0.5046
Encephalitis, Acute Disseminated Encephalomyelitis, Myelitis	3 (.241)	1 (.175)	1.0000	1 (196)	-	0.5355
Erythema Multiforme	1 (.080)	2 (0.350)	0.1776	-	2 (1.042)	0.0240
Fever	14 (1.123)	11 (1.926)	0.1704	8 (1.572)	4 (2.083)	0.6516
Guillain-Barre Syndrome	6 (.481)	-	0.0906	1 (0.196)	-	0.5355
Infective Abscess	1 (.080)	-	0.4498	-	-	-
Nodule	2 (0.160)	-	0.2850	-	-	-
Other Severe or Unusual Events	104 (8.340)	43 (7.531)	0.5607	29 (5.697)	11 (5.729)	1.0000
Pain and/or swelling	49 (3.929)	27 (4.729)	0.4275	35 (6.876)	15 (7.813)	0.6802
Rash	121 (9.703)	52 (9.107)	0.6857	52 (10.216)	11 (5.729)	0.0631
Severe Diarrhea and/or vomiting	124 (9.944)	56 (9.807)	0.9471	53 (10.413)	16 (8.333)	0.4052
Thrombocytopenia	8 (0.642)	4 (0.701)	0.8028	2 (0.393)	2 (1.042)	0.3446

N_1^{P} - Total Pfizer dose 1 administered; N_2^{P} - Total Pfizer dose $\geq$2 administered.

N_1^{M} - Total Moderna dose 1 administered; N_2^{P} - Total Moderna dose $\geq$2 administered.

(A)

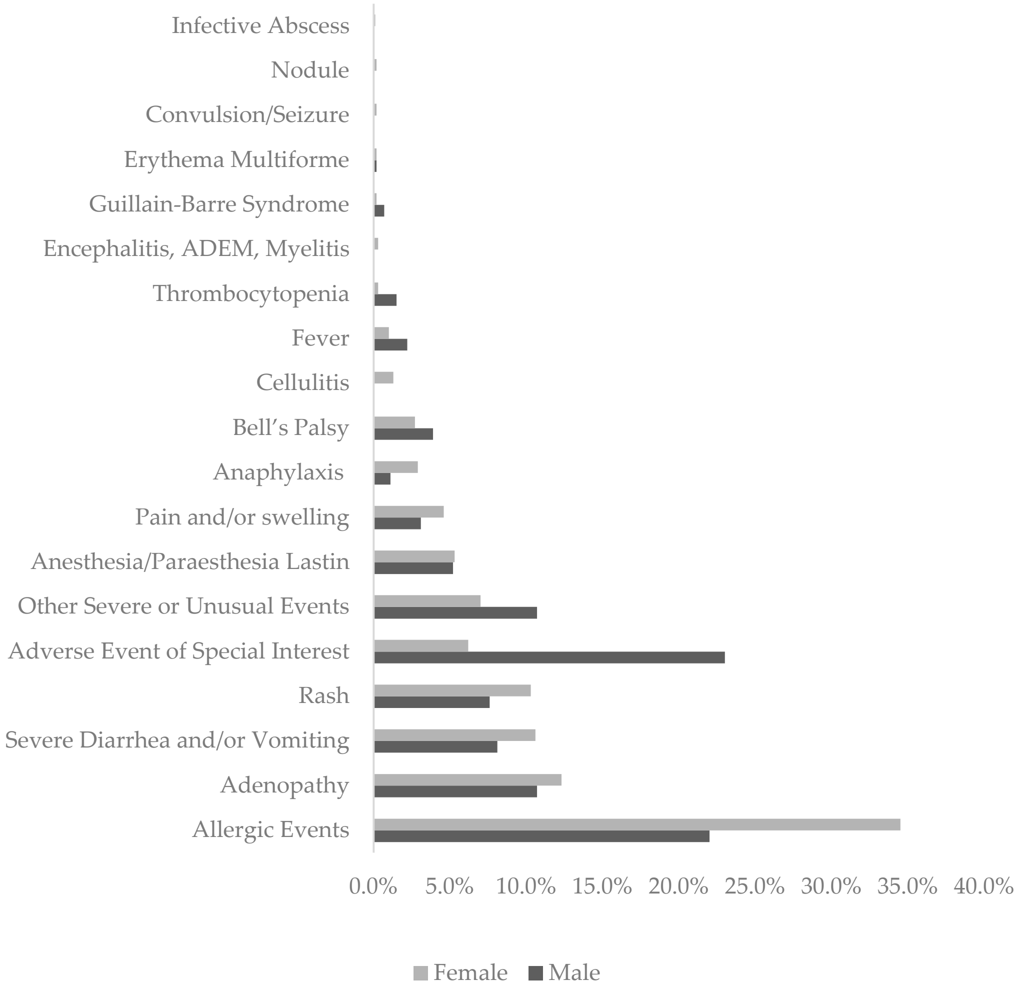

Infective Abscess
Nodule
Convulsion/Seizure
Erythema Multiforme
Guillain-Barre Syndrome
Encephalitis, ADEM, Myelitis
Thrombocytopenia
Fever
Cellulitis
Bell's Palsy
Anaphylaxis
Pain and/or swelling
Anesthesia/Paraesthesia Lastin
Other Severe or Unusual Events
Adverse Event of Special Interest
Rash
Severe Diarrhea and/or Vomiting
Adenopathy
Allergic Events
0.0% 5.0% 10.0% 15.0% 20.0% 25.0% 30.0% 35.0% 40.0%
Female
Male

(**B**)

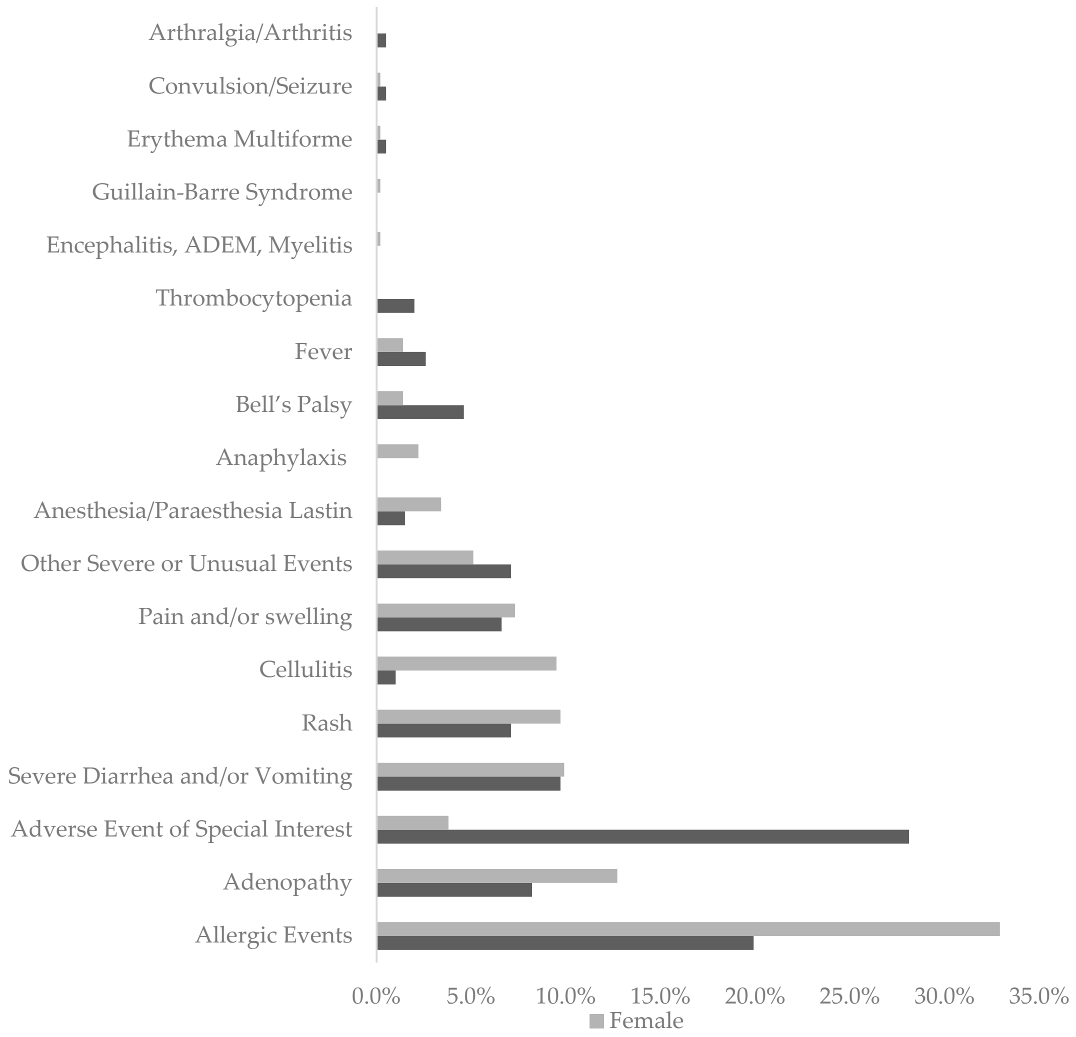

Figure S1. (**A**) AEFI type frequency by sex and by Pfizer, 14 December 2020 to 30 April 2022. (**B**) AEFI type frequency by sex and by Moderna, 14 December 2020 to 30 April 2022.
